# Abdominal manifestations of multisystem diseases: a pattern-based imaging review

**DOI:** 10.1007/s00261-026-05641-8

**Published:** 2026-06-26

**Authors:** Shayan Saeed, Jasmine Zhao, James Shi, Dillon Sommer, Samuel Mingo, Syed Ibrahim, Yusef Helmy, Vahid Yaghmai, Mohammad Helmy

**Affiliations:** Department of Radiological Science, Irvine Medical Center, University of California, Orange, USA

**Keywords:** IgG4-related disease, Behçet disease, Erdheim–Chester disease, Amyloidosis, Sarcoidosis, Multimodality imaging

## Abstract

Multisystem diseases frequently present with overlapping clinical and imaging manifestations, making diagnosis challenging and often delayed. Many of these entities involve the abdomen early in the disease course, and abdominal imaging may provide the first clue to a systemic process. A pattern-based imaging approach is therefore essential for narrowing the differential diagnosis and guiding further clinical evaluation. This review highlights the multimodality imaging features of several important multisystem inflammatory and infiltrative diseases encountered in abdominal radiology, including IgG4-related disease, Behçet disease, Erdheim–Chester disease, amyloidosis, and sarcoidosis. For each entity, key abdominal manifestations are emphasized alongside characteristic extra-abdominal imaging findings that may support the diagnosis. Imaging appearances with an emphasis on abdominal findings across computed tomography, magnetic resonance imaging, ultrasound, and ^18^F-fluorodeoxyglucose positron emission tomography/computed tomography are discussed with attention to disease distribution, enhancement characteristics, associated systemic findings, and common mimics. Although imaging findings are often nonspecific in isolation, integration of abdominal imaging patterns with extra-abdominal features can substantially narrow the differential diagnosis. Familiarity with these characteristic imaging manifestations may improve diagnostic confidence, guide targeted biopsy, and facilitate timely multidisciplinary management.

## Introduction

Multisystem diseases comprise a diverse set of disorders that share overlapping clinical features across multiple organ systems. Conditions like IgG4-related disease (IgG4-RD), Behçet disease, Erdheim–Chester disease, sarcoidosis, and systemic amyloidosis represent unique pathologies but often present with nonspecific manifestations, making clinical and radiologic differentiation challenging.

**Fig. 1:**
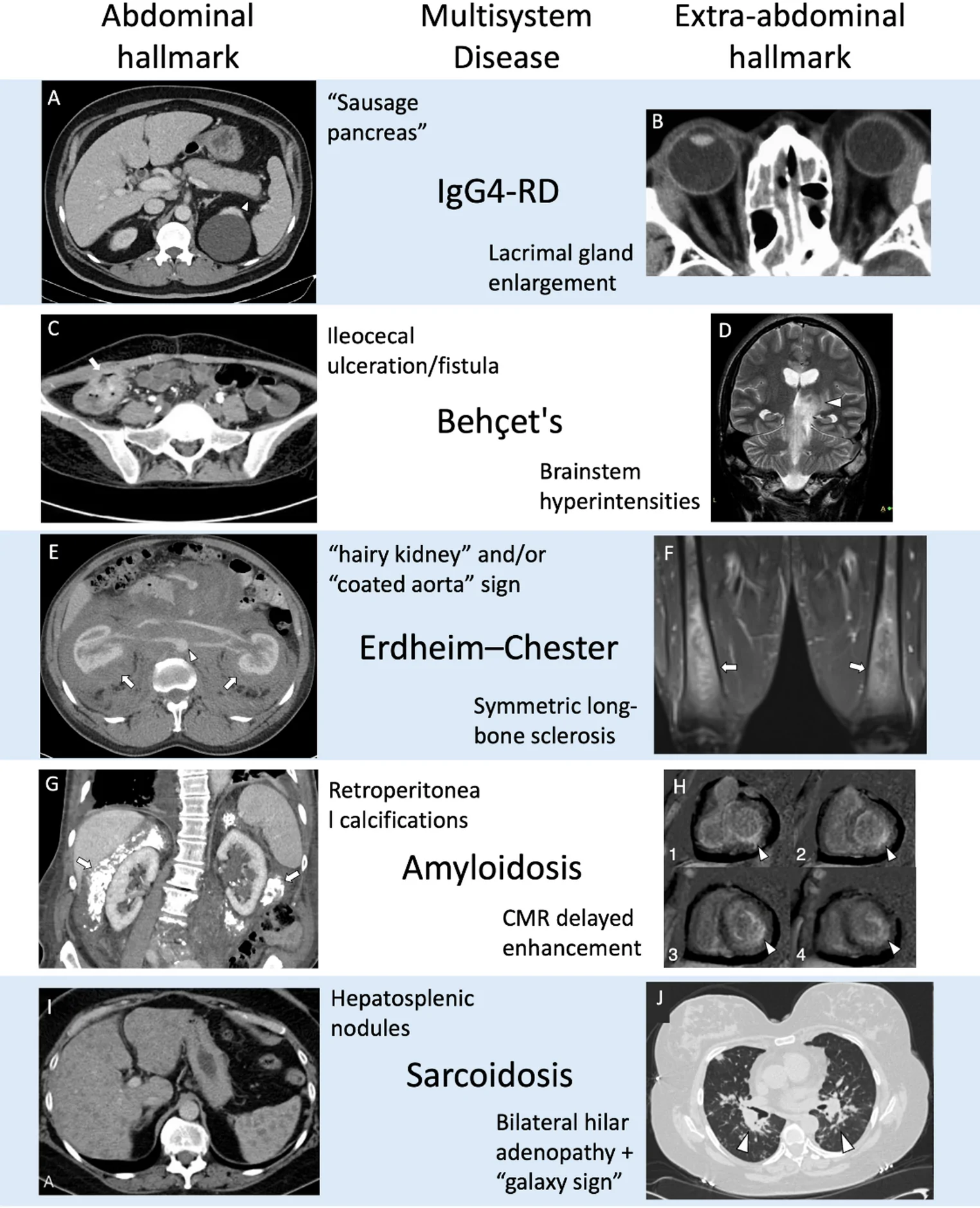
Pattern-based imaging manifestations of multisystem diseases. IgG4-RD demonstrating (**A**) autoimmune pancreatitis with diffuse pancreatic enlargement (“sausage pancreas”) and retroperitoneal fibrosis (white arrowhead) and **B** lacrimal gland enlargement. Behçet disease with **C** penetrating ileocecal inflammation (white arrow) and **D** hyperintense lesion in the left thalamus (white arrowhead) and brainstem. Erdheim–Chester disease **E** with perinephric soft tissue infiltration (“hairy kidney”) (white arrows), peri-aortic encasement (“coated aorta”) (white arrowhead), and **F** enhancement of the femur metadiaphyses (white arrows). Amyloidosis with **G** retroperitoneal calcifications (white arrow) and (H) cardiac MRI showing delayed gadolinium enhancement of the mitral annulus, mitral valve, and subvalvular apparatus (arrowheads) from amyloid deposition. Sarcoidosis with **I** hypoattenuating nodules in the liver and spleen and **J** bilateral perihilar conglomerates of perilymphatic pulmonary nodules (“galaxy sign”) (white arrowheads). Panels B, C, D, and I adapted from Kubota and Mortiani [1], He and Wu [2], Pretorius et al. [3], and Peters et al. [4], respectively, all under CC BY 4.0

Diagnostic imaging frequently provides the first evidence of systemic involvement and is essential for identifying, characterizing, and monitoring these diseases. However, overlapping abdominal manifestations and variable extra-abdominal involvement can complicate diagnosis. This review summarizes the clinical and pathophysiologic features of these diseases and highlights characteristic imaging findings, particularly in the abdomen, that may aid in their distinction from common mimics. Because imaging findings are frequently nonspecific in isolation, recognition of characteristic patterns of organ distribution and associated extra-abdominal manifestations is often the key to diagnosis (Fig. 1).

## IgG4-related disease (IgG4-RD)

### Clinical overview

Immunoglobulin G4-related disease (IgG4-RD) is a systemic, immune-mediated disorder characterized by tumefactive lesions and fibrosis of unknown etiology [5]. Commonly affected organs include the biliary tree, pancreas, kidneys, blood vessels, salivary glands, and orbital adnexal structures [6]. Unlike many autoimmune diseases, IgG4-RD shows a male predominance, particularly in older patients, who more frequently exhibit internal organ involvement [7]. Clinically, IgG4-RD often follows an indolent course with heterogeneous manifestations that can resemble a range of rheumatologic disorders. First-line treatment consists of systemic corticosteroids, with rituximab reserved for refractory or relapsing disease [6].

### Pathophysiologic basis

IgG4-RD is characterized by a dense lymphoplasmacytic inflammatory infiltrate composed of T and B lymphocytes, eosinophils, and plasma cells, often with extranodal germinal center formation [8]. From the earliest stages of the disease, a unique arrangement of spindle cells buried within the lymphoplasmacytic infiltrate produces a characteristic storiform-type pattern of fibrosis that often appears homogeneous and relatively hypoenhancing in early phases with progressive delayed enhancement on computed tomography (CT) and magnetic resonance imaging (MRI) [8]. Obliterative phlebitis is another key histopathologic feature of IgG4-RD and may involve small-, medium-, and large-caliber vessels [9].

### Diagnostic criteria and the role of imaging

Diagnosis of IgG4-RD requires integration of clinical, serologic, radiologic, and pathologic findings. Imaging plays a central role by identifying characteristic patterns of multiorgan involvement, assessing disease extent, guiding biopsy, and excluding important mimics. Although histopathologic confirmation remains the reference standard, the 2019 ACR/EULAR classification criteria recognize that characteristic imaging findings may contribute substantially to diagnosis, and classification may be achieved in selected cases without tissue confirmation or elevated serum IgG4 levels [10, 11].

### Key abdominal imaging findings

The most common sites of abdominal involvement include the pancreas and biliary tree followed by the aorta/retroperitoneum and kidneys [12].

#### Pancreatic involvement

**Fig. 2:**
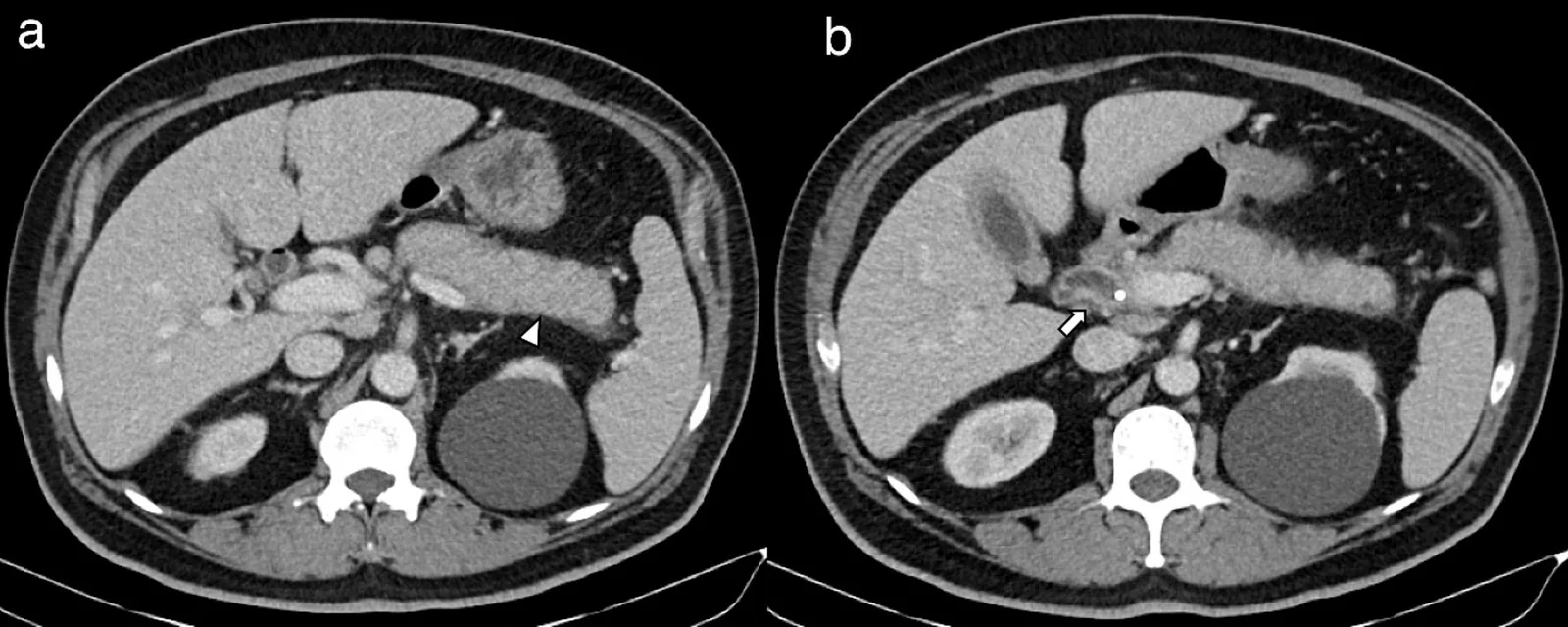
Axial CT images of the abdomen with **A** “sausage shape” appearance of the pancreas with loss of fat clefts, diffuse enlargement, and mild peri-pancreatic stranding (white arrowhead). **B** Diffuse thickening of the gallbladder and common bile duct walls with biliary stent in place compatible with biliary duct involvement

IgG4-RD is the most common cause of autoimmune pancreatitis (AIP), also classified as type 1 AIP. Imaging features include diffuse, smooth pancreatic enlargement (Fig. 2), resulting in a characteristic sausage-shaped appearance (“sausage pancreas”) with effacement of normal lobulations and a surrounding hypoattenuating capsule-like rim (“halo sign”) [13]. Less typical presentations include focal or multifocal pancreatic enlargement resembling an ill-defined mass. These atypical forms, which can mimic pancreatic cancer, frequently feature irregular narrowing of the main pancreatic duct and may be accompanied by a capsule-like rim or autoimmune-related extrapancreatic findings [13].

#### Biliary involvement

Biliary involvement, termed IgG4-related sclerosing cholangitis (IgG4-SC), occurs in up to 90% of patients with AIP, making bile ducts the second most common site of involvement [14]. The presence of pancreatic involvement can therefore help discriminate IgG4-SC from primary sclerosing cholangitis and cholangiocarcinoma [12]. Imaging findings include symmetric wall thickening and luminal narrowing with smooth inner and outer margins of the bile ducts, most commonly the intrapancreatic bile duct segment [15]. In 25% of patients with IgG4-SC and concomitant AIP, diffuse acalculous cholecystitis may also be present [16].

#### Renal involvement

**Fig. 3:**
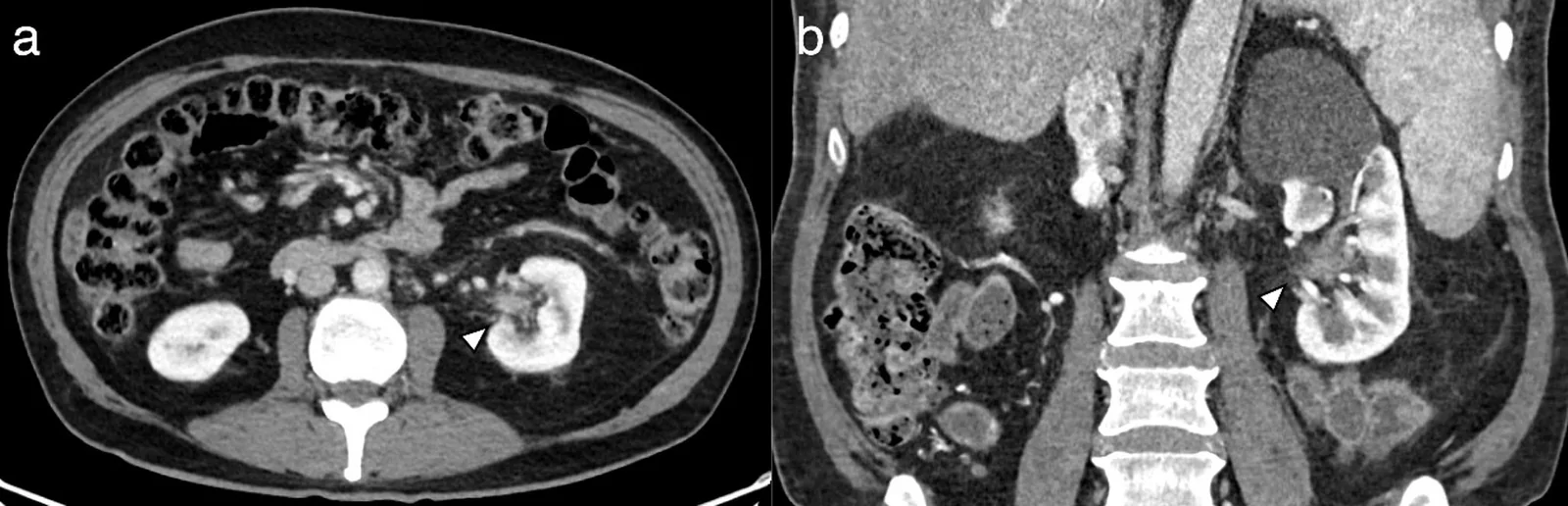
Axial (**a**) and Coronal (**b**) CT images of the abdomen and pelvis with soft tissue thickening surrounding the left renal pelvis (white arrowheads)

IgG4-related renal disease most commonly affects the renal parenchyma, typically manifesting as multiple small cortical wedge-shaped lesions (Fig. 3). Solitary IgG4-RD mass-like lesions are rarer and may mimic neoplasm [17]. On contrast-enhanced CT, lesions are hypodense in the arterial phase with progressive delayed enhancement [17]. MRI improves detection, with lesions appearing T2 hypointense relative to normal renal parenchyma.

#### Retroperitoneal and mesenteric involvement

**Fig. 4:**
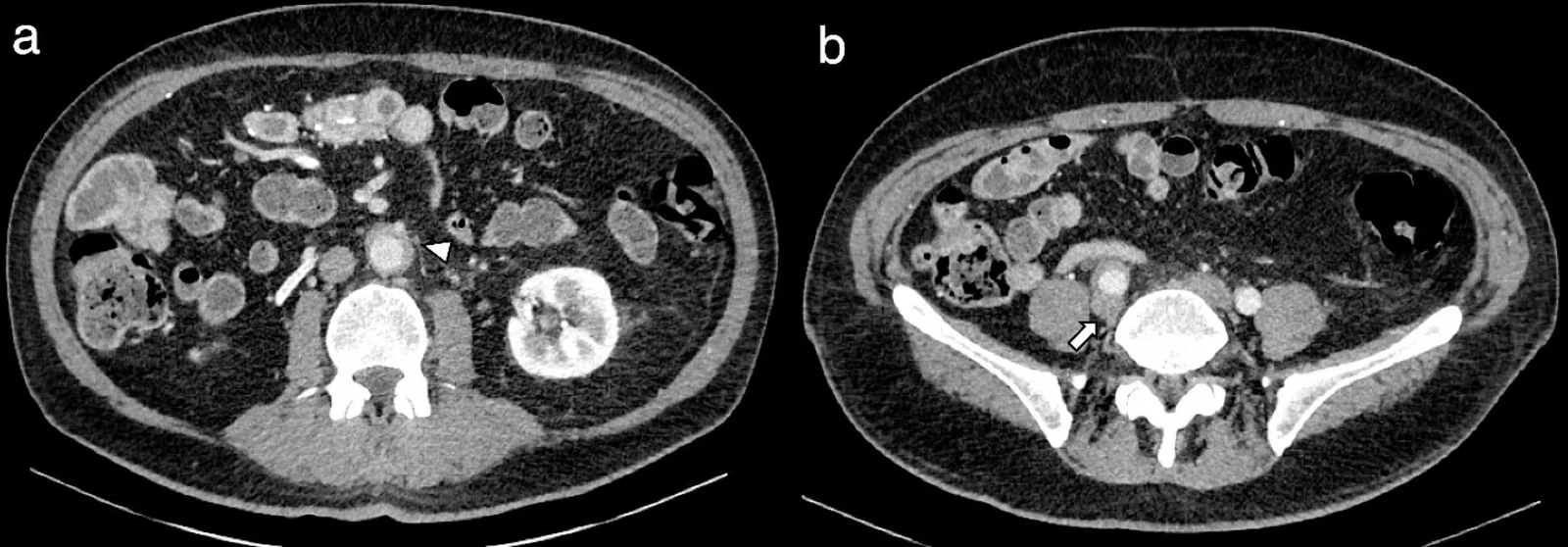
Axial CT images of the abdomen and pelvis with **A** soft tissue thickening surrounding the abdominal aorta (white arrowhead) and **B** the right common iliac artery (white arrow)

Retroperitoneal disease most often manifests as retroperitoneal fibrosis (RPF) and less commonly as peri-aortitis or inflammatory aneurysmal dilation [18]. On CT, RPF appears as homogeneous periaortic soft tissue below the renal pedicles, often extending to the common iliac arteries without aortic displacement or suprarenal extension, which helps distinguish it from lymphoma [19] (Fig. 4). Active disease can encase the ureters or involve the renal hila, leading to hydronephrosis [18].

MRI further characterizes extent and activity, with active RPF demonstrating T2 hyperintensity, contrast enhancement, and restricted diffusion. Diffusion-weighted imaging may be particularly useful when contrast is contraindicated [20].

### Cross-system imaging clues

#### Thoracic involvement

Up to half of patients with IgG4-RD will have thoracic involvement. Chest CT is the primary imaging modality and demonstrates four major pulmonary patterns including: solid nodular, round ground-glass opacity, alveolar interstitial, and bronchovascular. These findings are nonspecific and may mimic primary lung cancer or chronic interstitial lung disease. Reported prevalence of each pattern varies across studies, likely reflecting differences in patient populations and imaging interpretation. Extrapulmonary findings include mediastinal and hilar lymphadenopathy [21].

#### Salivary gland involvement

IgG4-related sialadenitis (IgG4-RS) occurs in up to 40% of patients with IgG4-RD and typically presents with bilateral salivary gland enlargement (historically termed Mikulicz disease), although isolated submandibular involvement (Kuttner pseudotumor) may be seen [12, 22]. Relative parotid sparing may help distinguish IgG4-RD from Sjögren syndrome. Ultrasound is the preferred initial imaging modality, demonstrating hypoechoic nodules with intervening hyperechoic septa [23]. CT and MRI are less sensitive for subtle nodularity, while ^18^F-FDG PET/CT may show nonspecific hypermetabolism [23].

#### Orbits involvement

**Fig. 5:**
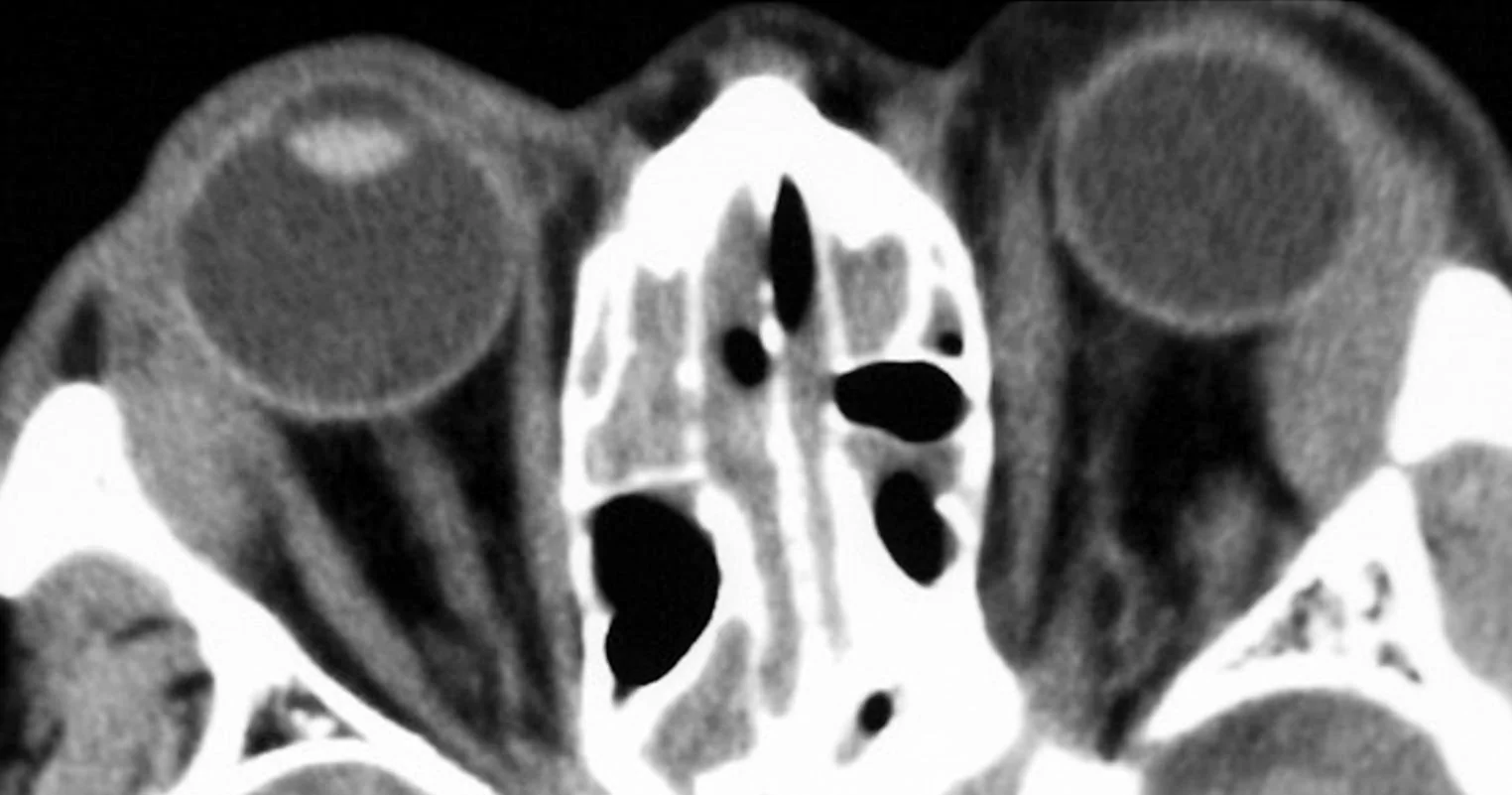
Axial CT of the head shows enlargement of the bilateral lacrimal glands in the setting of IgG4-RD. Reproduced from Kubota and Mortiani [1] under CC BY 4.0

Ophthalmic disease (IgG4-ROD) most commonly affects the lacrimal glands and extra-ocular muscles and less commonly peri-orbital fat [24] (Fig. 5). MRI typically shows homogeneous lacrimal gland enlargement with enhancement and relative T2 hypointensity reflecting fibrosis, while CT is useful for evaluating infiltration and osseous involvement [24]. In the absence of lacrimal gland disease, imaging may mimic thyroid-associated orbitopathy; however, predominant lateral rectus involvement—as opposed to inferior and medial rectus muscles—favors IgG4-ROD [25].

### Differential diagnosis and mimics

In the hepatobiliary system, IgG4-related disease may mimic primary sclerosing cholangitis or cholangiocarcinoma; however, long-segment smooth biliary strictures, frequent involvement of the intrapancreatic bile duct, and coexistent autoimmune pancreatitis favor IgG4-related sclerosing cholangitis [12]. In the pancreas, focal or diffuse IgG4-related pancreatitis can resemble pancreatic adenocarcinoma, but diffuse enlargement, a capsule-like rim, and lack of marked upstream pancreatic duct dilatation are more characteristic of IgG4-RD [13]. Renal involvement may mimic renal cell carcinoma, lymphoma, or urothelial carcinoma, although bilateral or multifocal cortical lesions with delayed enhancement and relative sparing of the collecting system support IgG4-related kidney disease [17]. Retroperitoneal fibrosis in IgG4-RD may also resemble lymphoma; however, the soft tissue typically encases rather than displaces the aorta and usually spares extension above the renal hila [19].

Teaching Points.

- **Immunoglobulin G4-related disease** (IgG4-RD) is **a fibro-inflammatory condition** of unknown etiology. Within the abdomen, the most affected organs include the **pancreas**,** biliary tree**,** kidneys**,** and aorta/retroperitoneum.**
- **IgG4-RD** is the most common cause of **autoimmune pancreatitis**. Look for the characteristic sausage-shaped appearance (“**sausage pancreas**”) with **effacement of normal lobulations** and a surrounding **hypoattenuating capsule-like rim** (“**halo sign**”). Cross-system imaging findings such as enlargement of the **salivary or lacrimal gland** or **pulmonary nodules** may be further clues.
- IgG4-RD lesions have a broad differential when considered in isolation. The pattern of involvement is key to early diagnosis and treatment with systemic corticosteroids.

## Behçet disease

### Clinical overview

Behçet disease is a chronic, relapsing, multisystem inflammatory disorder of unclear etiology characterized by the classic triad of recurrent oral aphthous ulcers, genital ulcers, and uveitis [26]. Epidemiologically, while the disease has a worldwide distribution, the highest prevalence is clustered along the ancient “Silk Road” extending from the Middle East to East Asia, with Turkey reporting up to 4 cases per 1,000 population [27]. The condition predominantly affects young adults between the ages of 20 and 40 and while gender distribution varies geographically, males often experience a more severe disease course with higher rates of major organ involvement and mortality [28]. Management is primarily immunosuppressive, with corticosteroids used for acute disease and biologic agents such as anti-TNF therapies for severe vascular or neurologic involvement [27].

### Pathophysiologic basis

Behçet is a variable vessel vasculitis affecting arteries and veins of any caliber, with venous involvement comprising approximately 85% of vascular manifestations. Pathogenesis involves environmental triggers acting on genetic predispositions that lead to hypercoagulability and endothelial dysfunction [28]. A neutrophil-predominant inflammatory infiltrate releases reactive oxygen species and neutrophil extracellular traps, driving thrombosis. This innate response is amplified by Th1, Th17, and cytotoxic T cells accumulating in perivascular spaces. In arteries, this inflammation destroys vasa vasorum elastic fibers, causing vessel dilation, aneurysms, and catastrophic rupture risk [27].

### Diagnostic criteria and the role of imaging

Because there are no pathognomonic laboratory tests or specific autoantibodies, the diagnosis of Behçet disease relies primarily on clinical evaluation using the diagnostic criteria established by the International Criteria for Behçet’s Disease (ICBD) [26]. The ICBD utilizes a weighted scoring system where oral aphthosis, genital aphthosis, and ocular lesions each receive 2 points, while skin lesions, neurological manifestations, and vascular manifestations each receive 1 point; a total score of 4 or more indicates a diagnosis of Behçet disease. Imaging plays a critical role in this diagnostic framework by detecting and characterizing major organ involvement that directly contributes to the diagnostic score and helps rule out mimicking conditions.

### Key abdominal imaging findings

#### Gastrointestinal involvement

**Fig. 6:**
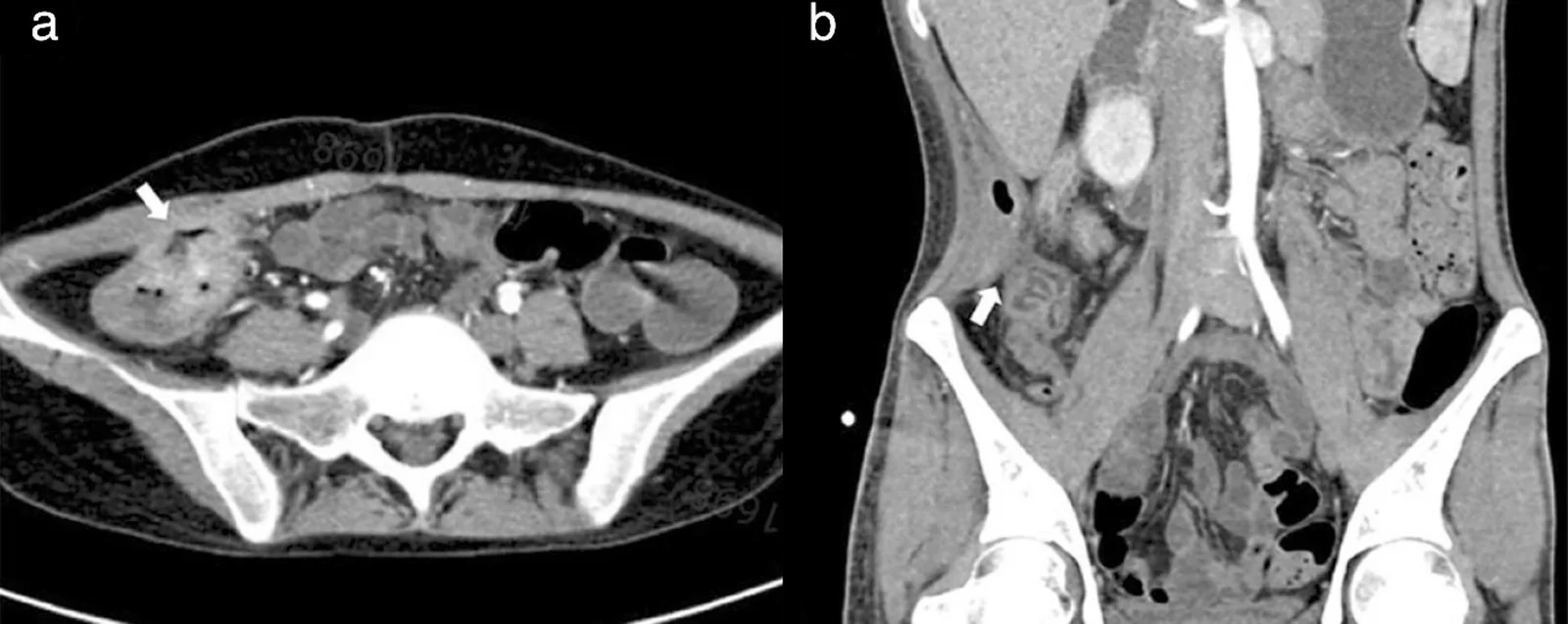
Axial (**a**) and coronal (**b**) reformat contrast-enhanced CT of the abdomen demonstrates mural thickening of ileocecal area with luminal stenosis and mural hyperenhancement (arrow, image a) and intestinal-abdominal fistula and mural abscess (arrow, image b). Adapted from He and Wu [2] under CC BY 4.0

Gastrointestinal involvement affects 10–50% of patients, typically at the terminal ileum and cecum [29]. CT hallmark findings include focal bowel wall thickening and deep, “volcano-shaped” penetrating ileocecal ulcerations (Fig. 6). Barium studies and CT may show thickened mucosal folds and polypoid lesions. These deep ulcers frequently cause severe complications like perforation, fistulas, and hemorrhage [29].

#### Abdominal vasculature involvement

Severe venous occlusions and arterial aneurysms are prominent. Venous thrombosis often affects the IVC, portal system, and hepatic veins; the latter can precipitate Budd-Chiari syndrome, showing delayed peripheral hepatic enhancement and a hypertrophied caudate lobe [29]. On the arterial side, the abdominal aorta and its branches are susceptible to saccular pseudoaneurysms due to vasa vasorum inflammation [27].

#### Hepatic and renal involvement

Primary parenchymal involvement is rare, but secondary congestive hepatosplenomegaly and portosystemic collaterals occur due to venous outflow obstruction [29]. Chronic systemic inflammation can rarely cause secondary AA amyloidosis, presenting as diffuse hepatic or renal enlargement [26].

### Cross-system imaging clues

#### Thoracic involvement

A leading cause of death is catastrophic hemoptysis from pulmonary artery aneurysms, best visualized on CT pulmonary angiography (CTPA). Superior vena cava thrombosis often causes mediastinal widening. Focal CT consolidations may represent active vasculitis or pulmonary infarction [30].

#### Peripheral vascular involvement

Lower extremity DVT and superficial thrombophlebitis are the most frequent vascular findings. Peripheral arterial pseudoaneurysms (e.g., femoral, popliteal) are less common but represent rupture-prone emergencies [27].

#### Central nervous system involvement

**Fig. 7:**
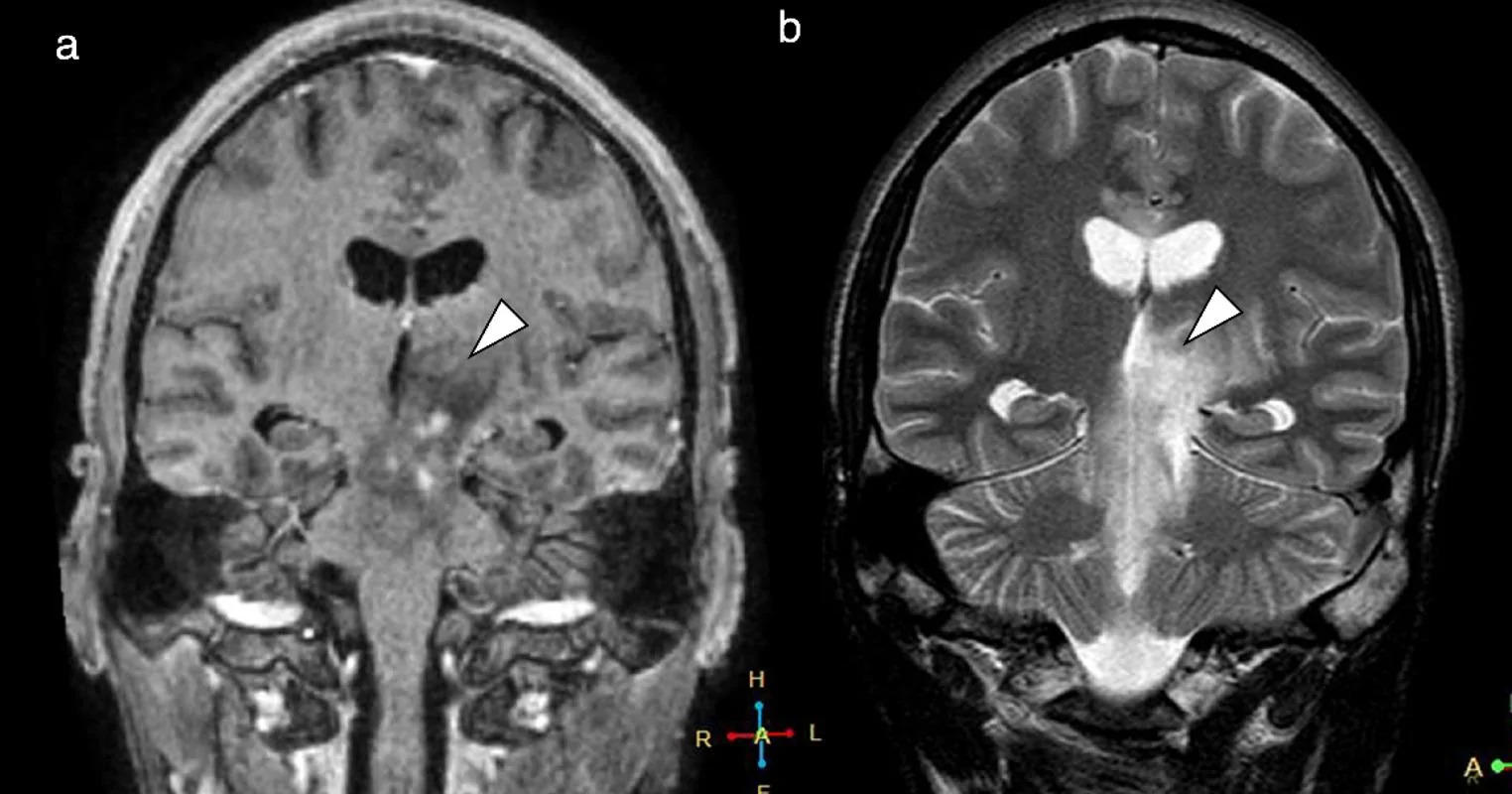
Coronal T1-weighted (**a**) and T2-weighted (**b**) images showing hyperintense signals in the left thalamus (white arrowheads), left midbrain, and bilateral pons. Adapted from Pretorius et al. [3] under CC BY 4.0

Neuro-Behçet predominantly affects small venules. MRI classically shows T2/FLAIR hyperintense lesions with perilesional edema favoring the ventral brainstem, basal ganglia, and mesodiencephalic junction [31]. Cerebral venous sinus thrombosis is also common, presenting as filling defects on MR or CT venography [27] (Fig. 7).

### Differential diagnosis and mimics

In the gastrointestinal tract, Behçet lesions mimic Crohn’s disease, but generally lack Crohn’s extensive stricturing and cobblestone mucosa [32]. Thoracic pulmonary artery aneurysms must be distinguished from Hughes-Stovin syndrome, which shares identical radiologic features but lacks classic mucocutaneous ulcers, leading many to consider it an “incomplete” Behçet variant [33]. In the brain, Neuro-Behçet mimics demyelinating diseases like multiple sclerosis; however, multiple sclerosis typically favors the dorsal brainstem and white matter tracts, whereas Behçet targets the ventral brainstem and mesodiencephalic junction [34].

Teaching Points.

- **Behçet disease** is a variable vessel **vasculitis** that uniquely presents with simultaneous **widespread venous thrombose**s and life-threatening **arterial aneurysms**.
- In the **gastrointestinal tract**, the presence of **deep**,** penetrating ileocecal ulcers** with **focal bowel thickening** should raise suspicion for Behçet disease and help **differentiate** it from **inflammatory bowel disease.**
- The combination of **pulmonary artery aneurysms** and **superior vena cava thrombosis** on chest imaging is highly characteristic and identifying it is critical to prevent fatal hemorrhagic complications.
- Diagnosis relies on the ICBD criteria, making a multidisciplinary imaging approach essential to capture the defining vascular, neurological, and gastrointestinal manifestations.

## Erdheim–Chester disease

### Clinical overview

Erdheim–Chester disease (ECD) is a rare multisystem non-Langerhans cell histiocytic neoplasm that typically affects adults and most commonly involves the skeleton, retroperitoneum, cardiovascular system, central nervous system, and orbits. Diagnosis is frequently delayed because the clinical manifestations are heterogeneous and nonspecific [35]. Radiology is key for this entity as characteristic combinations of osseous and extraosseous imaging findings may strongly suggest the diagnosis prior to tissue confirmation [36]. Treatment is driven by molecular profiling, with BRAF and MEK inhibitors used in patients harboring MAPK pathway alterations [35].

### Pathophysiologic basis

ECD is characterized by tissue infiltration by foamy histiocytes within a background of chronic inflammation and fibrosis. Molecular studies have established the disease as a clonal neoplasm driven by MAPK pathway alterations, most commonly *BRAF* V600E, with other MAPK pathway mutations also described. Histologically, lesions are composed of CD68-positive and CD1a-negative histiocytes, a pattern that helps distinguish ECD from Langerhans cell histiocytosis, although histopathologic findings may vary by site and can be subtle in some biopsies [37].

### Diagnostic criteria and the role of imaging

Diagnosis of ECD requires integration of radiologic, histopathologic, and molecular findings. Imaging is central to raising diagnostic suspicion and staging multisystem involvement, guiding biopsy to metabolically active or accessible lesions, and monitoring response to therapy [38]. In practice, the combination of a “hairy kidney” appearance, coated aorta, and bilateral symmetric long-bone sclerosis should strongly suggest ECD and prompt tissue sampling with molecular testing for MAPK pathway alterations [36].

### Key abdominal imaging findings

Within the abdomen, the most characteristic manifestations of ECD involve the retroperitoneum, kidneys, and aorta.

#### Retroperitoneal and renal involvement

**Fig. 8:**
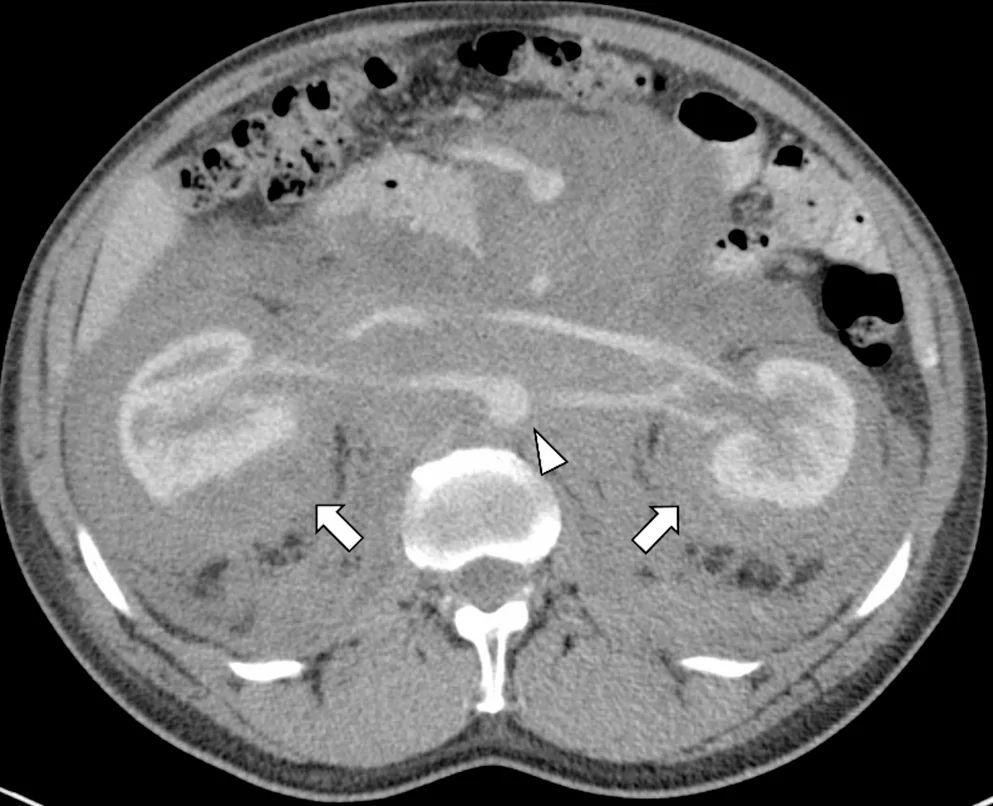
Axial CT image of the abdomen and pelvis with diffuse soft tissue infiltration of the bilateral perinephric fat (“hairy kidney sign”) (white arrow), abdominal aorta and its branches (“coated aorta sign”) (white arrowhead)

Approximately 50–70% of patients with ECD have retroperitoneal and renal involvement, with most being clinically asymptomatic. On CT, ECD in the retroperitoneum will appear as perinephric hypo-attenuated soft-tissue infiltrates with irregular contours and low contrast enhancement, producing the classic ‘hairy kidney’ sign (Fig. 8). On T1- and T2-weighted MRI, infiltrates appear isointense to muscle and exhibit enhancement after contrast administration. The disease may extend into the renal sinuses or proximal ureters, leading to hydronephrosis, post-renal obstruction, and eventual renal failure. In many cases, infiltrates may also spread to adrenal glands presenting as adrenal enlargements [39].

#### Aortic involvement

ECD may also produce plaque-like peri-aortic soft tissue encasing the abdominal aorta and branch vessels (termed the “coated aorta” sign) resulting in vascular complications (Fig. 8). Histiocytic infiltration into renal arteries may lead to decreased renal perfusion and eventual nephro-vascular hypertension requiring ureteral stenting. Notably, the pelvic structures including the pelvic ureters and lower urinary tract are spared in ECD [39].

### Cross-system imaging clues

#### Thoracic involvement

Pulmonary involvement is visualized with thoracic CT and is seen in nearly 50% of patients, manifesting as nodular interlobular septal thickening and consolidation in the upper lobes. Some patients may also demonstrate pleural effusion [40].

#### Cardiovascular involvement

Nearly half of patients with ECD have cardiac involvement most commonly in the form of a focal mural mass (pseudotumor) in the right atrium and atrioventricular sulcus [36]. On CT, the infiltrate appears as a soft-tissue mass with mild enhancement and isoattenuating relative to the myocardium. Vascular ECD manifests as circumferential perivascular infiltration, most commonly of the aorta (“coated aorta”). Compression of branch vessels—often the coronary and renal artery—may lead to downstream ischemia [36].

#### Skeletal involvement

**Fig. 9:**
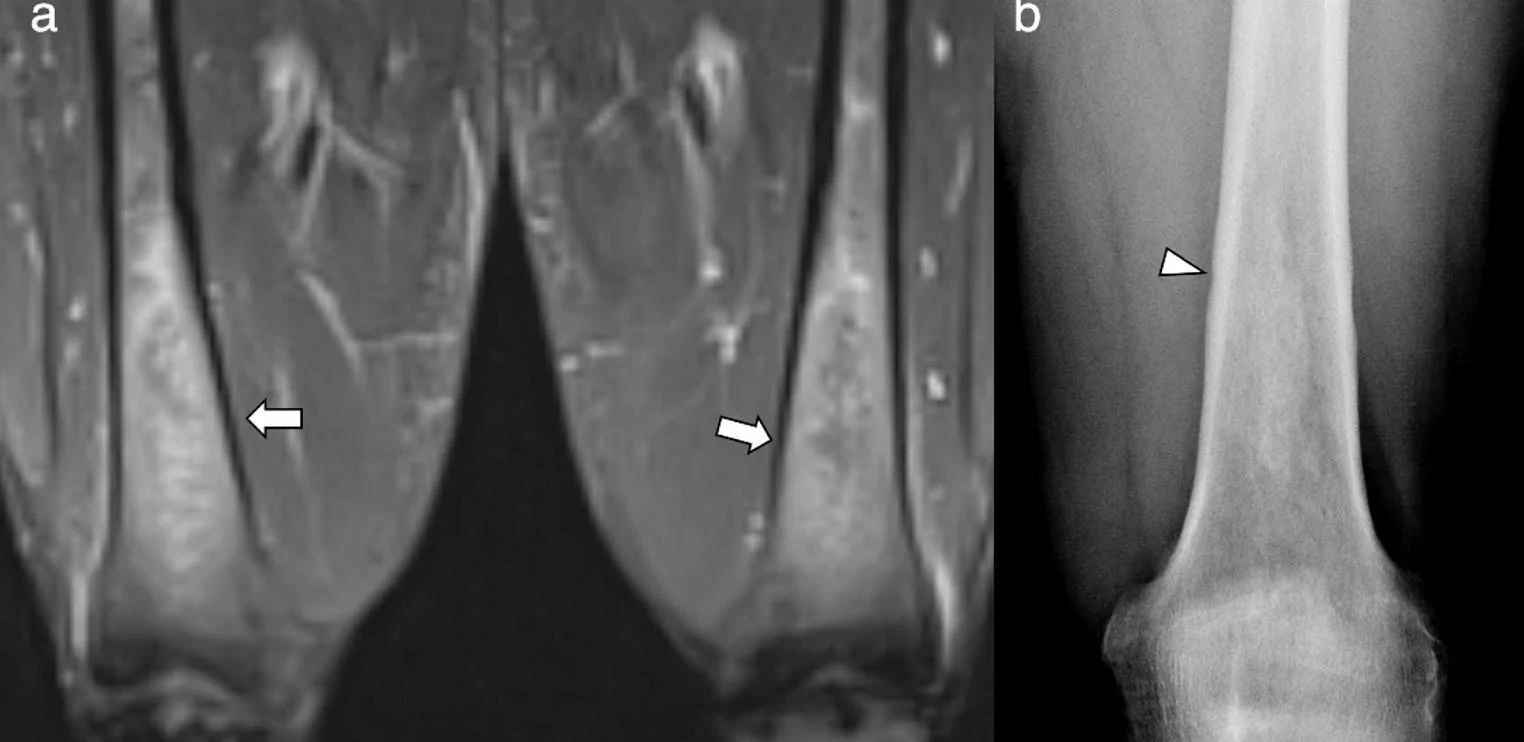
Coronal MRI (**a**) of the bilateral femurs with symmetric patchy enhancement of the distal metadiaphyses (white arrows) compatible with the histiocytic infiltrates. Radiograph of distal femur (**b**) shows sclerosis at the metadiaphysis (white arrowhead)

Skeletal involvement is the hallmark manifestation of ECD, occurring in nearly 95% of patients. Lower extremities and pelvis involvement is especially common, in addition to the clavicle, scapula, sternum, ribs, and facial bones. In cases of femur and tibial lesions, typical T2-weighted MRI features involve symmetric diffuse hyperintensity from osteosclerosis of the meta-diaphyseal regions with sparing of the subchondral bone, a pattern virtually pathognomonic for ECD (Fig. 9) [41]. Lesions will also show increased uptake at skeletal scintigraphy and ^18^F-FDG PET [36].

#### Intracranial involvement

**Fig. 10:**
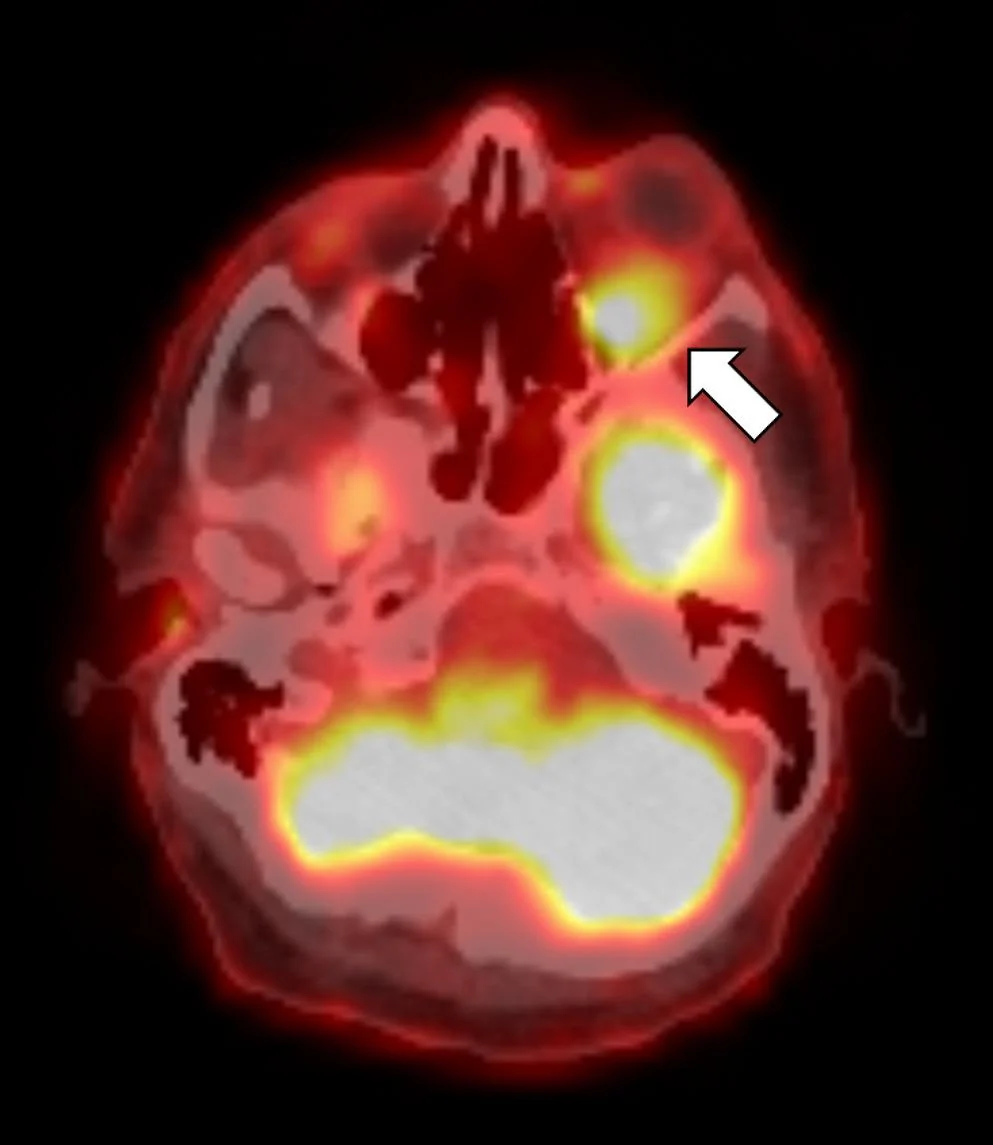
Axial ^18^F-FDG PET/CT image of the head with radiotracer uptake in the left retro-orbital soft tissue infiltration (white arrow)

Central nervous system and orbital involvement may also be present, including pituitary or hypothalamic lesions causing diabetes insipidus and retro-orbital soft tissue infiltration [36] (Fig. 10). Pituitary involvement most commonly manifests as loss of the normal T1-hyperintense posterior pituitary bright spot, often accompanied by pituitary stalk thickening or enlargement [42]. Orbital disease typically demonstrates T1- and T2-hypointense signal intensity with gadolinium enhancement and FDG-avid lesions [42].

### Differential diagnosis and mimics

The principal abdominal differentials include idiopathic retroperitoneal fibrosis, IgG4-RD, lymphoma, and large-vessel vasculitis. Compared with idiopathic retroperitoneal fibrosis, ECD more often centers at the renal hila and perirenal spaces and is more likely to involve the proximal ureters and perinephric fat. IgG4-RD may also produce retroperitoneal fibrosis and peri-aortic soft tissue, but the presence of characteristic long-bone sclerosis, right-sided cardiac involvement, or typical orbital disease favors ECD. Lymphoma may produce bulky retroperitoneal soft tissue or nodal disease, whereas vasculitis more commonly demonstrates mural rather than predominantly peri-adventitial aortic involvement [36, 43].

Teaching Points.

- **Erdheim–Chester disease** is a rare multisystem **histiocytic neoplasm** in which abdominal CT may first suggest the diagnosis through characteristic retroperitoneal findings.
- “**Hairy kidney**” and “**coated aorta**” are **classic imaging patterns** and should prompt evaluation for **skeletal**,** cardiovascular**,** CNS**,** and orbital involvement**.
- The pattern of multisystem disease, rather than any single lesion in isolation, is often the key to distinguishing ECD from IgG4-RD, retroperitoneal fibrosis, lymphoma, and vasculitis.

## Amyloidosis

### Clinical overview

Amyloidosis is a heterogeneous group of disorders characterized by extracellular deposition of misfolded amyloid proteins within tissues, resulting in progressive organ dysfunction. These deposits may occur in a systemic or localized distribution and are classified based on the precursor protein, including light-chain (AL), serum amyloid A (AA), transthyretin-related (ATTR), hereditary, dialysis-related, and localized forms. Clinical presentation is variable and depends on the organs involved, with common manifestations including restrictive cardiomyopathy, nephrotic syndrome, hepatomegaly, neuropathy, and constitutional symptoms [44].

Amyloidosis most commonly affects older adults, with a male predominance and peak incidence after the sixth decade of life. AL amyloidosis is the most frequently diagnosed systemic form in many clinical settings with some estimates suggesting roughly 1,275 to 3,200 new cases annually in the United States [45]. Therapy is subtype-dependent, with AL amyloidosis treated using plasma cell–directed regimens (including proteasome inhibitors and anti-CD38 agents) and ATTR amyloidosis managed with transthyretin stabilizers or gene-silencing therapies [44].

### Pathophysiologic basis

Amyloidosis is characterized by extracellular deposition of insoluble fibrillar proteins arranged in a cross–β-pleated sheet configuration. These deposits disrupt normal tissue architecture and lead to progressive organ dysfunction. Histologically, amyloid demonstrates Congo red positivity with characteristic apple-green birefringence under polarized light [46].

The distribution and imaging appearance are influenced by the underlying amyloid subtype. AL amyloidosis is commonly associated with plasma cell dyscrasias and frequently demonstrates multisystem involvement, whereas ATTR amyloidosis shows a predilection for cardiac involvement [46].

### Diagnostic criteria and the role of imaging

Diagnosis of amyloidosis requires a multidisciplinary approach integrating clinical, laboratory, imaging, and histopathologic findings. Tissue biopsy with Congo red staining remains the gold standard for definitive diagnosis with subtyping identified by immunohistochemistry or mass spectrometry. Multimodal imaging does not establish amyloidosis by itself, but is crucial for detecting disease extent, guiding biopsy, and raising suspicion.

### Key abdominal imaging findings

The most commonly affected abdominal organs include the liver, spleen, kidneys, and retroperitoneum; however, involvement is highly variable and often nonspecific [47].

#### Hepatic and splenic involvement

**Fig. 11:**
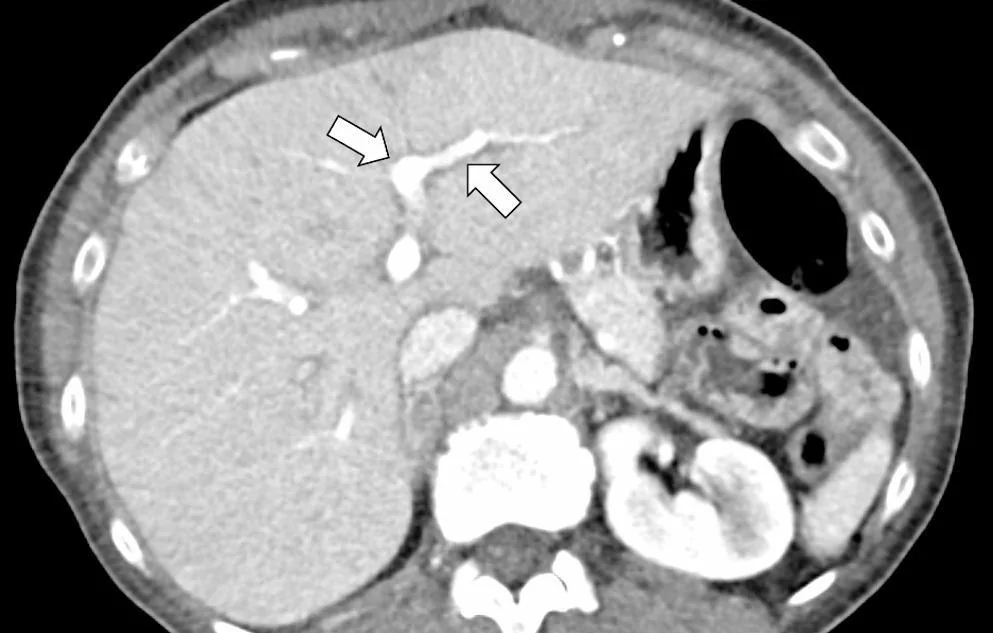
Axial contrast enhanced CT of the abdomen with liver heterogeneity and mild periportal thickening (white arrow)

Hepatic and splenic amyloidosis typically presents as hepatomegaly, heterogeneous attenuation or enhancement, periportal infiltration, or periportal thickening (Fig. 11). Splenic involvement can produce diffuse low T2 signal intensity on MRI; in some cases, the spleen also shows T1 signal abnormalities or calcification [47].

#### Renal involvement

Renal amyloidosis most commonly manifests as bilateral kidney enlargement in early disease, but as small, contracted kidneys with cortical thinning in established systemic disease. MRI and CT can show heterogeneous renal parenchyma with reduced enhancement and sometimes calcifications or focal mass-like deposits [47].

#### Retroperitoneal and mesenteric involvement

**Fig. 12:**
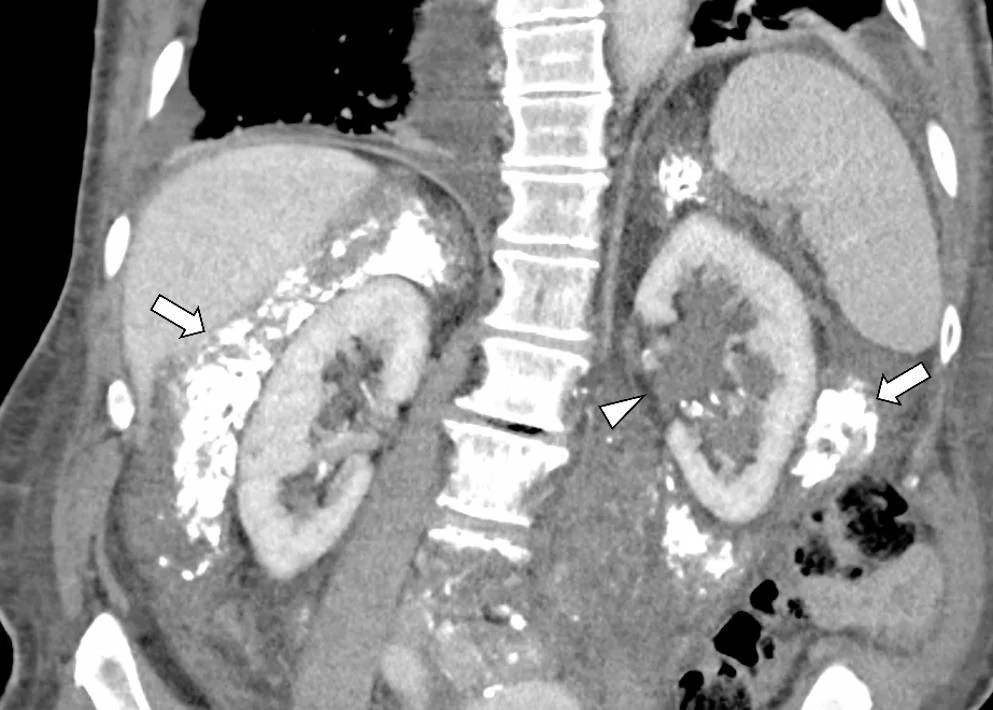
Coronal CT of the abdomen with retroperitoneal calcifications (white arrow) surrounding the bilateral kidneys and moderate left hydronephrosis (white arrowhead)

Amyloid deposition within the retroperitoneum may appear as infiltrative soft tissue with coarse calcifications, often encasing vascular structures such as the abdominal aorta and extending into the mesentery. This pattern may mimic retroperitoneal fibrosis or malignancy but tends to demonstrate a more diffuse infiltrative appearance with calcifications [48] (Fig. 12).

#### Cross-system imaging clues

##### Thoracic involvement

Pulmonary amyloidosis most commonly occurs in two forms: nodular parenchymal and diffuse alveolar septal. The former is more common and is often found incidentally on a chest X-ray in an asymptomatic adult. Nodules can occur in the airways, lung parenchyma, or pleura and demonstrate nonspecific imaging findings that may mimic other diseases. Symptomatic patients will likely have alveolar septal pulmonary amyloidosis. On CT, these appear as well-defined micronodules with accompanying interlobular septal thickening and reticular opacities. Airway disease will demonstrate tracheobronchial wall thickening or narrowing [48].

##### Cardiac involvement

**Fig. 13:**
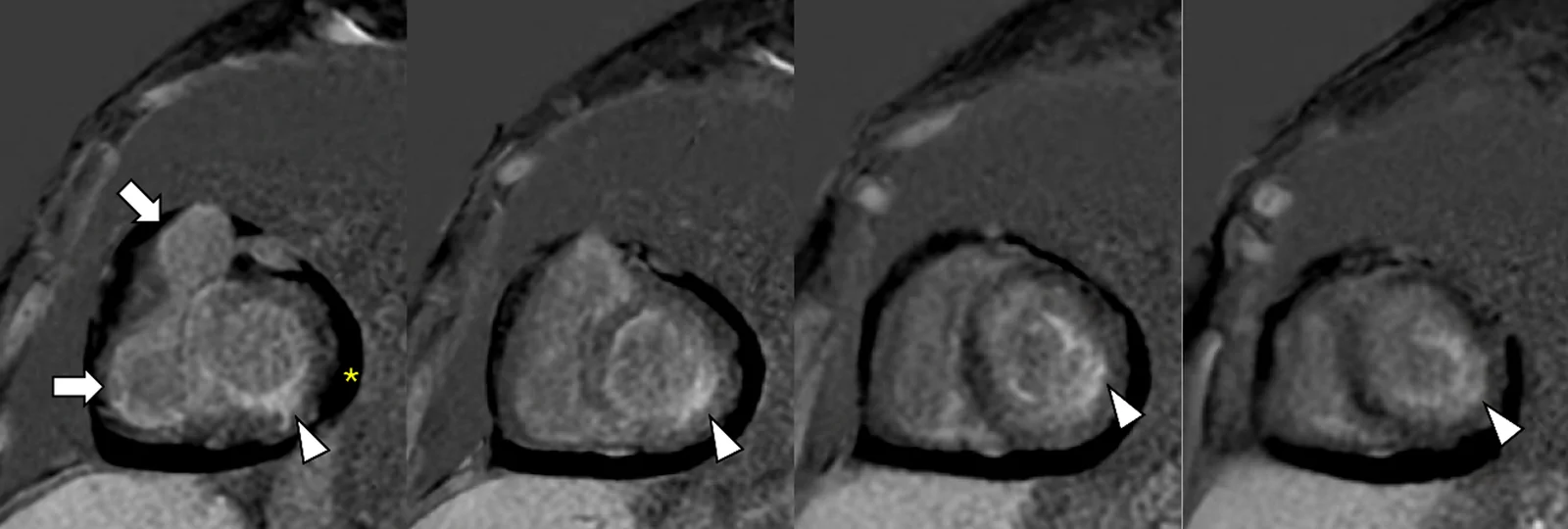
71-year-old female with cardiac MRI short axis views showing delayed gadolinium enhancement of the mitral annulus, mitral valve, and subvalvular apparatus (arrowheads). There is also mild diffuse subendocardial hyperenhancement (arrows), which is suggestive of amyloid infiltration, and a small pericardial effusion (asterisk)

Echocardiography is often the initial screening examination for cardiac involvement, whereas cardiac MRI (CMR) is a valuable tool in excluding amyloidosis in suspected cases and for differentiating it from other infiltrative cardiomyopathies. Regarding the subtypes of cardiac amyloidosis, CMR is still not considered a reliable standalone test, but several signal characteristics may help to differentiate between them. AL amyloidosis is marked by the presence of concentric left ventricular wall thickening while ATTR commonly shows asymmetric septal hypertrophy and more pronounced biventricular systolic dysfunction [49]. Diffuse subendocardial late gadolinium enhancements (LGE) without transmural extension is a pattern more frequently associated with AL amyloidosis [50] (Fig. 13). LGE pattern of ATTR amyloidosis frequently involves the right ventricular free wall and shows an initial basal predilection, progressing to biventricular diffuse transmural extension in advanced disease [50]. Additionally, native T1 is higher in AL amyloidosis, while ECV is higher in ATTR [51]. AL amyloidosis also shows greater T2 values reflecting the myocardial edema from direct light chain cardiotoxicity [52]. For patients who cannot undergo CMR, dual-energy CT (DECT) may represent an emerging alternative for detecting amyloidosis. Extracellular volume (ECV) quantification is the most validated CT application and shows strong diagnostic performance and correlates strongly with CMR-ECV [53, 54]. Elevated myocardial ECV reflects expansion of the extracellular space by amyloid deposits and correlates with disease burden.

### Differential diagnosis and mimics

Amyloidosis may mimic a variety of disease processes depending on the organ system involved. Pulmonary nodules may resemble metastatic disease or granulomatous infection, while interstitial patterns can overlap with fibrotic lung disease. Cardiac involvement may mimic hypertrophic cardiomyopathy or hypertensive heart disease, although diffuse subendocardial enhancement and apical sparing favor amyloidosis. In the abdomen, diffuse organomegaly without discrete mass favors infiltrative processes such as amyloidosis over malignancy. Retroperitoneal involvement may resemble lymphoma or retroperitoneal fibrosis but is more likely to demonstrate calcifications and infiltrative encasement of structures [44, 48].

Teaching Points.

- **Amyloidosis** is a **protein-misfolding disease** with **extracellular fibril deposition** and progressive organ dysfunction.
- AL amyloidosis is driven by a monoclonal plasma cell disorder and usually requires biopsy plus amyloid typing for diagnosis.
- Imaging is most **useful** for **detecting organ involvement** and **subtype differentiation**, especially in the heart and abdomen, but it is rarely specific enough alone for amyloidosis.
- Abdominal clues include **heterogeneous liver appearance**, **periportal infiltration**, **diffuse low T2 splenic signal**, and **smooth bowel wall/fold thickening**.
- Important mimics include ischemia, inflammation, lymphoma, carcinoma, iron overload, and hypertrophic cardiomyopathy depending on the organ system.

## Sarcoidosis

### Clinical overview

Sarcoidosis is an idiopathic multisystem granulomatous disease characterized by non-caseating granulomas that coalesce into nodules and masses. Incidence ranges from 2 to 60 per 100,000 worldwide, highest in Scandinavian countries and the United States. It predominantly affects women and patients under 40, with a second peak in women over 50. Treatment is typically corticosteroid-based, with steroid-sparing immunosuppressants (e.g., methotrexate or azathioprine) used in chronic or refractory disease [55].

### Pathophysiologic basis

Sarcoidosis likely represents an exaggerated cell-mediated immune response in genetically predisposed individuals [55]. The hallmark non-caseating granuloma, composed of epithelioid histiocytes, multinucleated giant cells, lymphocytes, and fibroblasts, may progressively fibrose, causing organ dysfunction [56].

### Diagnostic criteria and the role of imaging

Diagnosis of sarcoidosis is based on clinical history, histopathologic evidence of noncaseating granulomas, and consistent imaging findings. Imaging is central to identifying characteristic patterns of thoracic and extrathoracic involvement, and radiologist play an important role in assessing disease progression, selecting biopsy targets, and evaluating treatment response. In many patients, the combination of bilateral hilar and mediastinal lymphadenopathy with perilymphatic pulmonary nodules provides the initial clue to the diagnosis [57].

### Key abdominal imaging features

The most commonly affected abdominal organs by sarcoidosis are the liver, spleen, and kidneys. In most cases, abdominal involvement occurs in association with pulmonary disease and uncommonly as an isolated finding.

#### Hepatic and splenic involvement

**Fig. 14:**
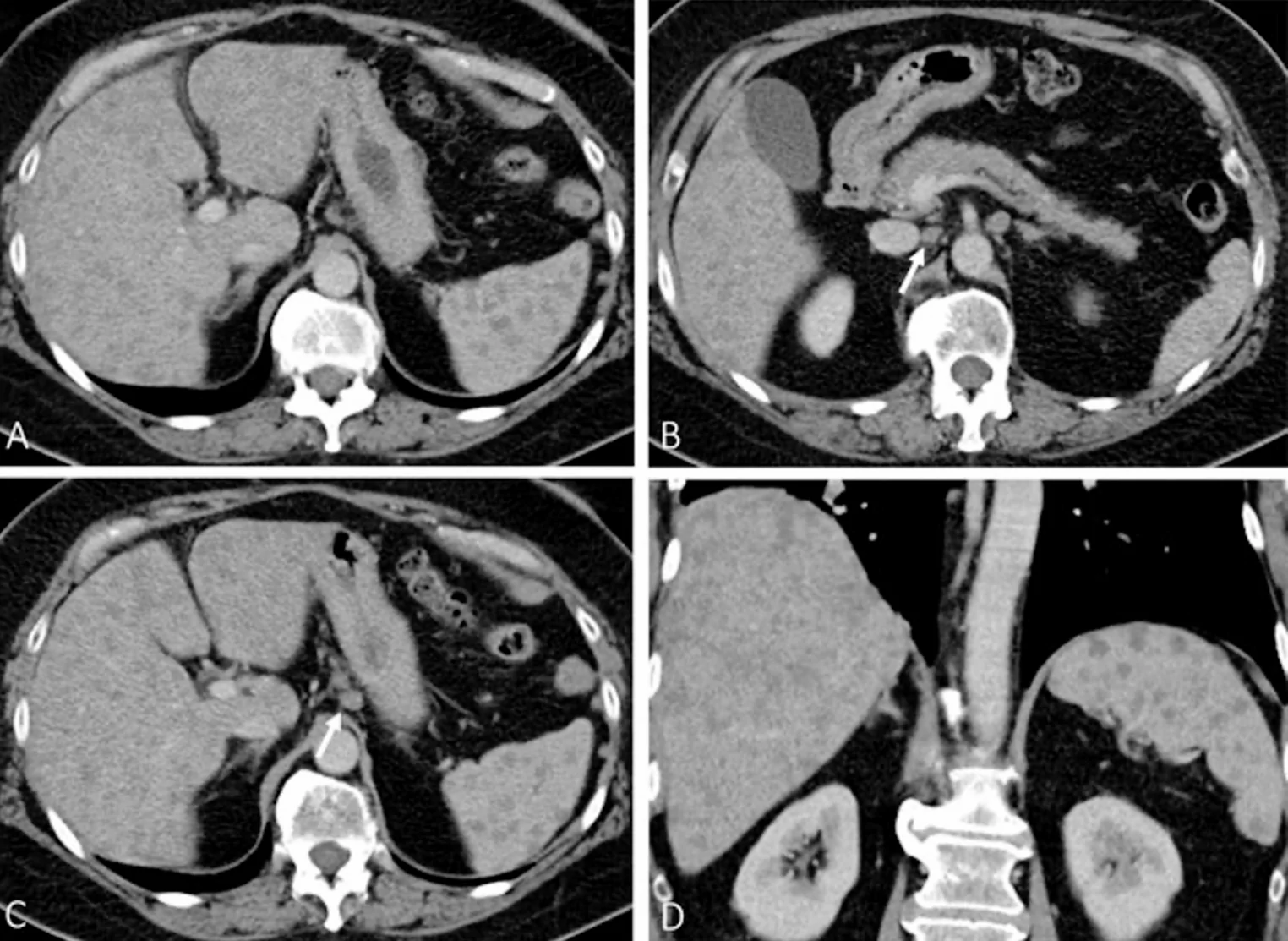
Axial (**A**–**C**) and coronal (**D**) CT abdomen in the poral venous phase. **A** shows numerous subcentimeter hypoattenuating nodules in the liver and spleen. **B** and **C** demonstrate lymphadenopathy (white arrows) in the retroperitoneum and along the lesser curvature of the stomach. **D** Mild hepatomegaly. Reproduced from Peters et al. [4] under CC BY 4.0

Hepatic and splenic involvement is common but often subclinical. The most common radiographic finding of hepatic sarcoidosis is hepatomegaly and while the liver appears homogeneous in most cases, some patients may show hepatic granulomas along the portal triads. These appear as hypoechoic on ultrasound, hypoattenuating on CT, and T1/T2-hypointense with hypoenhancement on MRI. Small granulomas may coalesce into macroscopically visible lesions that are diffusely distributed. Splenic involvement demonstrates similar imaging characteristics with splenomegaly and coalescent nodules [58] (Fig. 14). ^18^F-FDG PET/CT may help distinguish active granulomatous inflammation from fibrotic or quiescent disease. The most common findings include diffuse homogenous FDG uptake throughout the liver and/or spleen, reflecting widespread granulomatous infiltration. Focal or multifocal FDG-avid nodules may also be seen on FDG-PET, but these uptakes may mimic metastatic disease or lymphoma [59]. Furthermore, ^18^F-FDG PET/CT may be used to assess response to glucocorticoid therapy and to guide biopsy site selection, demonstrating a sensitivity of 92% in one series [60].

#### Biliary involvement

Sarcoidosis involvement of the intra-hepatic biliary ducts will often take the form of granuloma formation in the portal triad that may closely mimic primary biliary cirrhosis with the only differentiating feature being the absence of antimitochondrial antibodies. Extra-hepatic duct involvement will produce strictures with resultant cholestasis that mimics cholangiocarcinoma [58].

#### Renal involvement

Renal sarcoidosis manifests as interstitial nephritis and glomerulonephritis, with CT findings of striated nephrograms, nephromegaly, or low-attenuation nodules. Associated hypercalcemia leads to nephrocalcinosis and nephrolithiasis [61].

### Cross-system imaging clues

#### Thoracic involvement

**Fig. 15:**
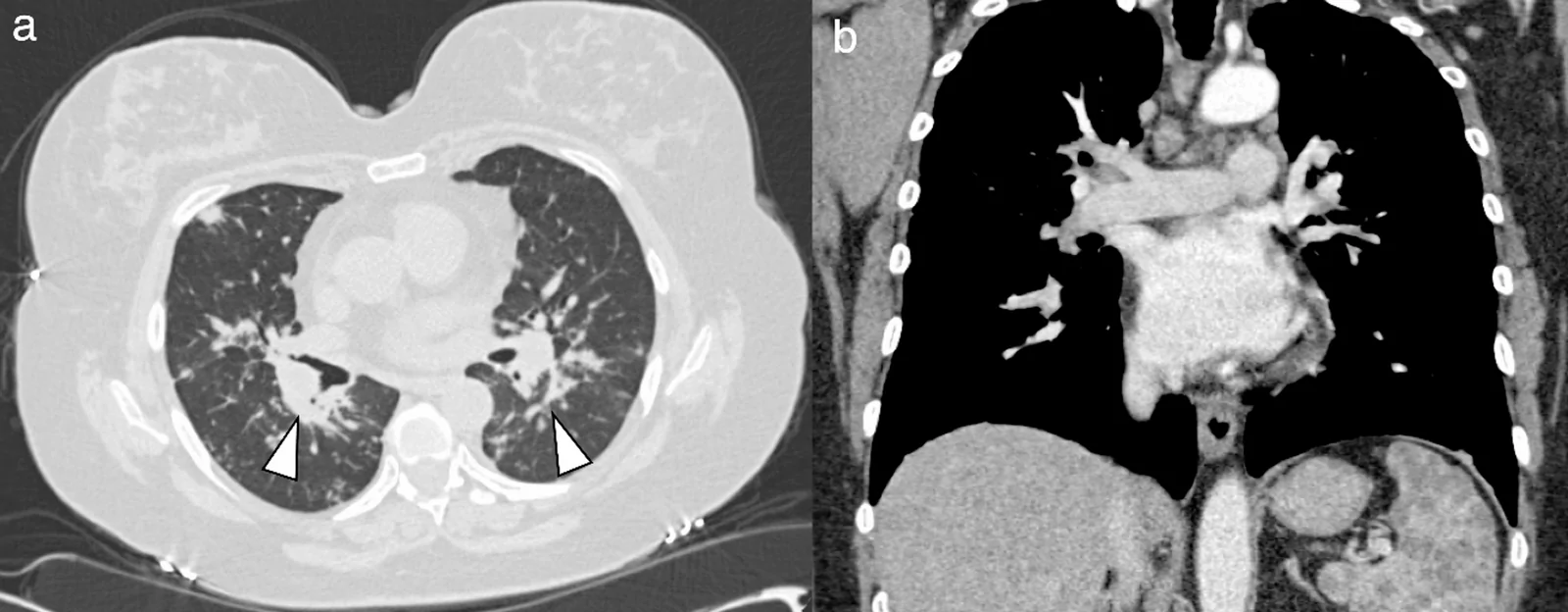
Axial CT chest (**a**) with bilateral, symmetric perihilar consolidations and scattered micronodules along the bronchovascular bundles, producing the characteristic “galaxy sign” (white arrowheads). Coronal CT chest (**b**) with mediastinal lymphadenopathy (white arrowheads) with mild calcifications

Thoracic involvement is the most common manifestation of sarcoidosis seen in over 90% of patients [62]. Initial evaluation of pulmonary sarcoidosis is classified using the four-stage Scadding criteria based on fibrotic findings from chest X-ray. However, high-resolution computed tomography (HRCT) demonstrates greater sensitivity than chest X-ray and is preferred for detecting imaging features [63]. The most typical finding is enlargement of bilateral hilar and right paratracheal lymph nodes, termed the Garland triad. Interstitial lung disease involves perilymphatic micronodules (1–3 mm in diameter) in the mid and upper lung zones that correspond to aggregates of epithelioid granulomas. Predilection for the axial interstitium is the classic case of pulmonary sarcoidosis but nodules may be diffusely distributed throughout the lung [64]. Granulomas may occasionally coalesce to form larger nodules manifesting in a pattern of consolidation with irregular margins. Micronodules may cluster around a larger central nodule giving the so-called ‘galaxy sign’ [65] (Fig. 15).

#### Cardiac involvement

Cardiac involvement is a common manifestation in patients with sarcoidosis. Approximately 5% of patients will have clinical symptoms while 20–25% have cardiac sarcoidosis that is clinically silent [66]. Imaging assessment with CMR reveals late gadolinium enhancement due to granulomatous inflammation or fibrosis in the myocardium in a non-vascular pattern while sparing the endocardium [67]. ^18^F-FDG PET may differentiate active cardiac sarcoidosis from inactive sarcoidosis or scarring from prior myocarditis, demonstrating focal or focal-on-diffuse patterns of hypermetabolic activity [67].

#### Central nervous system involvement

Neurosarcoidosis is a rare but life-threatening form of sarcoidosis seen in 5% of patients [68]. Cranial nerve palsies, especially of the facial and optic nerve, are the most common presentations followed by manifestation of parenchymal disease including seizures, focal deficit, and headache [68]. In suspected cases, contrast-enhanced MRI is the imaging modality of choice and typically demonstrates T2/FLAIR hyperintense lesions with homogeneous gadolinium enhancement. Common findings include pachymeningeal thickening and leptomeningeal enhancement [69].

### Differential diagnosis and mimics

The imaging appearance of sarcoidosis can overlap with a wide range of inflammatory, infectious, and neoplastic conditions, making clinicoradiologic correlation essential. Thoracic manifestations may mimic lymphoma, metastatic disease, pneumoconioses, hypersensitivity pneumonitis, and granulomatous infections [65]. In the abdomen, hepatic, splenic, and renal lesions can resemble lymphoma, leukemia, metastatic disease, disseminated infection, or IgG4-RD [70]. Cardiac sarcoidosis may mimic myocarditis or ischemic cardiomyopathy [67], while neurosarcoidosis can simulate leptomeningeal metastases, vasculitis, demyelinating disease, or chronic infectious meningitis [69]. Multisystem involvement, characteristic perilymphatic pulmonary nodules with symmetric hilar-mediastinal adenopathy, and concordant histopathology can help distinguish sarcoidosis from its mimics.

**Table 1:** Key imaging features of selected multisystem diseases with abdominal involvement

Disease	Key Abdominal Imaging Patterns	Characteristic Signs	Extra-abdominal Clues
IgG4-related disease	autoimmune pancreatitis, biliary involvement, renal lesions, retroperitoneal fibrosis	“sausage pancreas”, “halo sign”	salivary/lacrimal enlargement, thoracic lymphadenopathy
Behçet disease	Ileocecal ulcers, bowel thickening, venous thrombosis, aneurysms	“volcano-shaped ulcers”	pulmonary artery aneurysms, CNS lesions
Erdheim–Chester Disease	Perinephric infiltration, retroperitoneal fibrosis, peri-aortic disease	“hairy kidney”, “coated aorta”	long bone sclerosis, right atrial pseudotumor
Amyloidosis	Hepatosplenic involvement, renal changes, retroperitoneal calcification	none truly specific	cardiac amyloidosis, pulmonary disease
Sarcoidosis	Hepatosplenic nodules, renal involvement	“galaxy sign”	hilar + right paratracheal nodes

Teaching Points.

- **Sarcoidosis** is a multisystem **granulomatous disease** in which abdominal imaging may reveal **hepatosplenic** and **renal involvement** through characteristic **hypoattenuating nodules**,** organomegaly**,** and nephrocalcinosis.**
- **Bilateral hilar lymphadenopathy**,** perilymphatic micronodules**, and the “**galaxy sign**” on chest CT are **hallmark thoracic findings** that should prompt evaluation for extrathoracic disease.
- The pattern of multisystem granulomatous involvement, rather than any single organ finding, is key to distinguishing sarcoidosis from lymphoma, metastatic disease, disseminated infection, and IgG4-RD.

## Conclusion

Multisystem inflammatory and infiltrative diseases such as IgG4-RD, Behçet disease, Erdheim–Chester disease, amyloidosis, and sarcoidosis frequently involve the abdomen early in the disease course, and abdominal imaging may provide the first clue to a systemic process. While each entity has characteristic abdominal patterns — from the “sausage pancreas” of autoimmune pancreatitis to the “hairy kidney” of ECD — no single imaging feature is diagnostic in isolation, and considerable overlap exists among these diseases and their mimics, including lymphoma, malignancy, infection, and other forms of vasculitis and fibrosis. The key to narrowing the differential lies in recognizing the specific combination and distribution of organ involvement: for example, retroperitoneal fibrosis with pancreatic enlargement and lacrimal gland disease points toward IgG4-RD, whereas retroperitoneal infiltration with bilateral long-bone osteosclerosis and a right atrial pseudotumor is virtually pathognomonic for ECD. Integrating these organ-distribution patterns with cross-system imaging clues — particularly thoracic, cardiovascular, neurologic, and musculoskeletal findings — alongside clinical history, laboratory data, and histopathology enables radiologists to suggest the correct diagnosis earlier, guide targeted biopsy, and facilitate timely multidisciplinary management. Advances in targeted and disease-specific therapies—including corticosteroids and rituximab for IgG4-RD, biologic agents for Behçet disease, BRAF/MEK inhibitors for Erdheim–Chester disease, plasma cell- or transthyretin-directed therapies for amyloidosis, and immunosuppressive therapy for sarcoidosis—have further increased the importance of imaging for treatment selection and monitoring response. In sum, familiarity with these pattern-based distinctions across the discussed multisystem diseases is essential as the radiologist may be the first to connect disparate imaging findings into a unifying diagnosis (Table 1).

## Data Availability

No datasets were generated or analysed during the current study.
